# Antimicrobial Biomaterials Based on Composites of Metal Nanoparticles and Plant Extracts

**DOI:** 10.3390/ma18235474

**Published:** 2025-12-04

**Authors:** Assem Mukhtarkhanovna Kaliyeva, John G. Hardy

**Affiliations:** 1 Department of Biochemistry, Asfendiyarov Kazakh National Medical University, Almaty 050012, Kazakhstan; 2 Department of Chemistry, Lancaster University, Lancaster LA1 4YB, UK; j.g.hardy@lancaster.ac.uk

**Keywords:** antimicrobial biomaterials, biopolymer matrices, metal nanoparticles, innovative technologies

## Abstract

The global challenge of antimicrobial resistance, as well as the need to develop safe and environmentally sustainable materials, has served to stimulate research interest in antimicrobial technologies. The abundance, degradability and environmental friendliness of biopolymers means that they are widely used in medicine, pharmacy, and cosmetology. The focus of this mini review is the development of biopolymer matrices with antimicrobial properties imparted via the inclusion of metal nanoparticles and plant extracts. The review also examines innovative technologies, including photocatalytic systems and intelligent coatings with mechanisms for the controlled release of active substances that can be used to combat microbial infections. We believe that such materials have significant potential for eventual translation to products in the real world.

## 1. Introduction

According to the World Health Organization, antimicrobial resistance (AMR) is responsible for over 700,000 deaths annually worldwide, with projections suggesting this number could rise to 10 million by 2050 if no effective solutions are implemented [1]. The growing resistance of microorganisms to existing antibiotics poses a serious global challenge, leading to more complicated treatments, longer hospitalizations, and increased healthcare costs [2,3]. This urgent situation underscores the need for innovative strategies, including the development of biodegradable and environmentally friendly antimicrobial materials, which can help combat resistant pathogens while minimizing ecological impact [4,5,6,7]. These challenges have driven the search for environmentally friendly and multifunctional antimicrobial materials. In this context, multicomponent systems based on biopolymers have emerged as a promising strategy. By integrating biopolymer matrices with metal nanoparticles, plant-derived bioactive compounds, and stimuli-responsive or encapsulated delivery systems, it is possible to create “smart” materials with enhanced antimicrobial efficacy, controlled release of active agents, and adaptive responses to environmental stimuli. Such multifunctional systems offer effective, sustainable, and targeted solutions for medical devices, biomedical coatings, and other applications where reliable microbial control is essential [8,9,10,11,12,13,14,15,16].

Recent advancements in the development of antimicrobial materials focus on creating systems that combine biopolymers with metal-based nanoparticles (NPs) and plant extracts. These materials employ a multi-pronged approach to combat bacteria, including physical damage to cell walls, membrane disruption, generation of reactive oxygen species (ROS), and interference with cellular processes such as DNA and protein synthesis [17]. Key achievements include the development of “green synthesis” methods that use plant extracts to produce nanoparticles, functionalizing biopolymer matrices with nanoparticles to enhance mechanical and biological properties and improving efficacy against multidrug-resistant bacteria. Special attention is given to “green synthesis” of nanoparticles, where plant extracts serve as both antimicrobial agents and reducing and stabilizing agents for nanoparticle formation. These methods are significantly more environmentally friendly than traditional chemical synthesis [18,19,20,21,22,23,24,25].

Moreover, the integration of nanoparticles into biopolymer matrices allows for the controlled release of antibacterial agents, improving therapeutic efficacy and reducing side effects [26,27,28,29]. Examples of nanoparticles such as silver (Ag), gold (Au), zinc oxide (ZnO), and copper oxide (CuO) have already demonstrated high effectiveness against a wide range of bacteria, including multidrug-resistant strains. In multi-component nanoparticles, such as Au–Pt–Ag composites, these effects are enhanced through the combined targeting of multiple mechanisms [30,31,32,33,34].

Biopolymer matrices including nanoparticles stabilized by polymers can be further enriched with plant extracts, which contain flavonoids, terpenes, alkaloids, and other active compounds, contributing to the development of multifunctional biomaterials. These active substances have antiseptic and antioxidant properties [35,36,37]. The interaction of plant extracts with metal nanoparticles and polymers can significantly enhance the antimicrobial activity of the materials. For example, extracts of plants such as tea tree, turmeric, thyme and turnip are used as part of multicomponent systems to enhance their antimicrobial and antioxidant properties [38,39]. For example, the combination of silver nanoparticles (AgNPs) with chitosan improves the mechanical characteristics of the materials (i.e., shear strength and tensile strength) and increases antimicrobial activity [40,41,42]. The addition of plant extracts can increase a material’s antioxidant properties, which may help protect against degradation of the material under the influence of external factors such as ultraviolet radiation or oxygen [43,44,45,46,47]. For example, nanostructured titanium dioxide (TiO_2_), which exhibits strong photocatalytic and antimicrobial activity when exposed to ultraviolet or visible light. The generated reactive oxygen species (ROS) effectively disrupt bacterial membranes and biofilms, while the material itself remains chemically stable and cytocompatible. Furthermore, TiO_2_ nanoparticles can be synthesized via green chemistry approaches using plant extracts as natural reducing and stabilizing agents, minimizing the use of toxic reagents and enhancing the environmental safety of the process [48,49].

These materials find wide applications in healthcare (e.g., wound dressings, medical device coatings), food packaging, and other areas where controlling microbial growth is essential [50,51]. Thus, the creation of multicomponent systems, including metal nanoparticles, biopolymers and plant extracts, enables the development of effective and environmentally friendly antimicrobial materials. Such combined systems have potential for the creation of environmentally safe and effective antimicrobial materials.

Recent studies have demonstrated that these multicomponent biopolymer-based systems can be engineered to respond to specific environmental stimuli, such as pH changes, light, or the presence of enzymes [52]. For instance, nanoparticles coated with chitosan and hyaluronic acid selectively degrade in the acidic microenvironment of bacterial biofilms, facilitating penetration and eradication [53]. Similarly, a range of stimuli-responsive nanoplatforms has been developed to release antimicrobial agents in response to external cues, providing controlled and targeted antibacterial action [54].

Intelligent coatings that can control the release of active substances are exciting. These coatings can respond to changes in external factors such as pH, temperature, or the presence of pathogens [55,56,57,58]. Bacterial colonization and biofilm formation on medical devices continue to pose a major challenge in modern healthcare. Addressing this issue effectively requires the development of smart, personalized biomaterials tailored for use in medical device manufacturing. Particular focus is placed on surface modification strategies that inhibit bacterial adhesion and prevent the development of mature biofilms.

**Figure 1:**
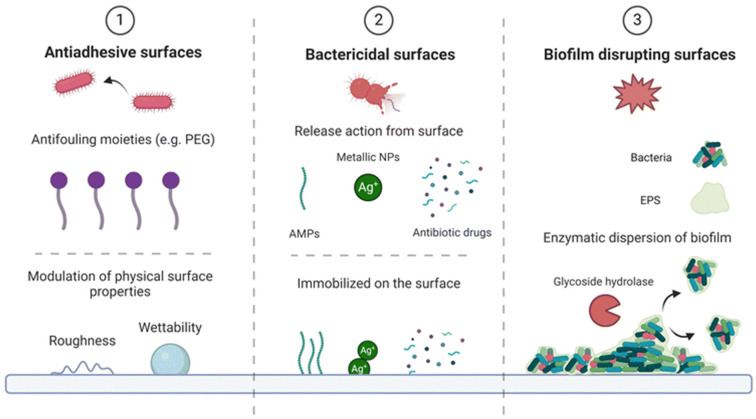
Scheme summarizing the three conventional defense mechanisms adopted in the design of (1) anti-adhesive surfaces, exploiting physical and chemical modifications, (2) bactericidal surfaces and (3) biofilm disrupting antimicrobial coatings that exploit immobilization or release of active molecules. The strategies and/or molecules mentioned are a few examples of the several options available in the literature [58]. Adapted from Cassa, M.A. et al. *Biomater. Sci.* 2024, 12, 5433–5449. https://doi.org/10.1039/D4BM00936C. © 2024, licensed under CC BY-NC 3.0. (https://creativecommons.org/licenses/by-nc/3.0/ accessed on 11 November 2025).

The authors of the review in [58] provide three bioinspired strategies for engineering antibacterial and anti-adhesive coatings that are capable of responding to external stimuli. Such stimulus-responsiveness enables coatings to exhibit controlled antibacterial activity that is selectively triggered in the presence of bacterial agents, thereby acting as a self-protective mechanism. Given that bacterial adhesion, proliferation, and subsequent colonization occur at the interface between microorganisms and medical devices, functionalizing biomaterial surfaces with antimicrobial properties presents a promising strategy to address this challenge. Various approaches can be employed to combat infection, targeting different stages of surface colonization and utilizing distinct mechanisms of action. Three such strategies are illustrated in Figure 1 [58].

Thus, the development of multicomponent antimicrobial systems based on biopolymers enables the creation of effective, environmentally friendly, and “smart” materials. In these systems, the biopolymer matrices provide a cytocompatible framework for stabilizing metal nanoparticles and incorporating biologically active plant-derived components, such as polyphenols and essential oils [59,60,61]. To enhance stability, bioavailability, and controlled release, active substances can be encapsulated in nano- or microcarriers, protecting them from oxidative degradation and ensuring targeted delivery. The integration of stimulus-responsive mechanisms allows these materials to function as intelligent coatings, releasing antimicrobial agents in response to changes in pH, temperature, or the presence of pathogens [62,63,64]. Additionally, the use of mechanical nanostructures that physically disrupt microorganisms contributes to the creation of adaptive and self-defensive surfaces [65,66]. These multifunctional systems improve the effectiveness against resistant bacteria and biofilms, offering sustainable and adaptable solutions for medical devices, biomedical coatings, and other applications where microbial control is critical [67,68]. The following sections of this article review current research and development in these areas, as well as the prospects for applying such systems to produce effective and environmentally sustainable antimicrobial materials.

Multifunctional and stimuli-responsive biopolymer-based systems represent the next generation of antimicrobial materials [69,70,71,72]. By combining biopolymer matrices with metal nanoparticles [73], plant-derived bioactive compounds [74], and smart delivery mechanisms, these systems bridge biotechnology and materials science, offering enhanced antimicrobial efficacy, targeted action, and adaptive responses to environmental stimuli.

## 2. Methodology

**Figure 2:**
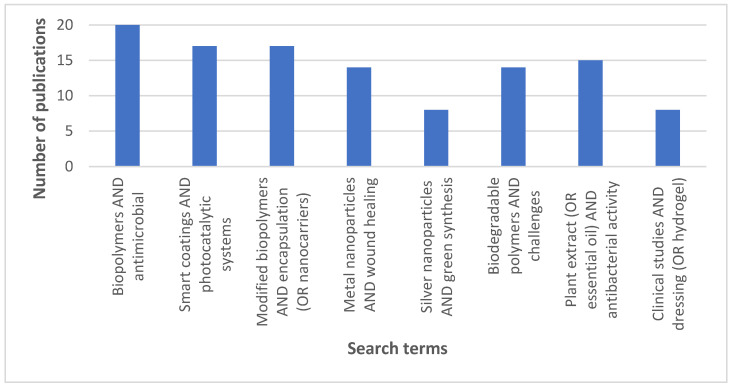
Number of publications retrieved from Scopus for different keyword combinations used in the literature search.

A literature search was performed in leading international scientific databases, including PubMed, Scopus, and Web of Science, covering publications from 2015 to 2025. A predefined set of keywords and logical operators (*AND*, *OR*) was applied to identify the most relevant studies on the development of antimicrobial biopolymer matrices. Key search terms included: biopolymer matrix, antimicrobial, metal nanoparticles, plant extract, smart coatings, photocatalytic systems, and their various combinations (Figure 2). Selection criteria focused on original research reporting experimental results, as well as review articles providing structured analyses of current trends and approaches.

The selected studies were analyzed comparatively, with attention to methodological approaches, material compositions, reported antimicrobial performance and geographical reach (ensuring inclusion of research from authors from the global north and south). This approach allowed for an overview of the literature, facilitating observation of patterns, innovative strategies, and emerging directions in the field of antimicrobial bionanocomposite materials.

**Figure 3:**
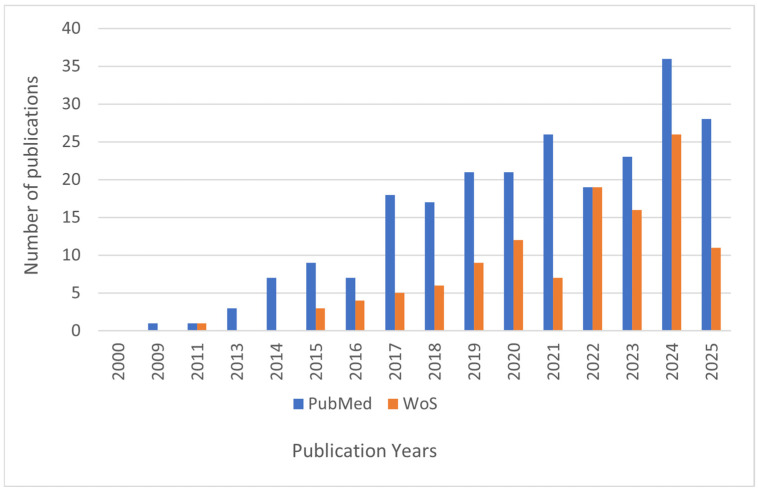
Annual number of publications (2000–2025) on antimicrobial composites based on metal nanoparticles and plant extracts indexed in PubMed (blue bars) and Web of Science (WoS, orange bars). The data show a significant increase in research interest over time, especially after 2013.

Figure 3 shows the annual number of publications from 2000 to 2025 related to antimicrobial composites based on metal nanoparticles and plant extracts, according to searches in two major scientific databases, PubMed and Web of Science (WoS). The data reveal a clear upward trend in research activity in this field over the last decade, reflecting growing scientific interest and recognition of the potential of these materials.

Publications indexed in PubMed demonstrate a steady increase starting around 2013, with a notable surge after 2017. Similarly, WoS publications show a consistent growth pattern, though in smaller absolute numbers compared to PubMed, indicating complementary but distinct indexing scopes.

This increasing volume of research underscores the expanding exploration of multifunctional antimicrobial composites, highlighting the integration of metal nanoparticles and plant extracts as promising strategies to address antimicrobial resistance. The upward trend also suggests the maturation of this interdisciplinary field, combining nanotechnology, biotechnology, and materials science.

The Cochrane review findings indicate that the success of new health technologies in laboratory settings often does not translate into real-world effectiveness [75,76,77]. Key barriers include weak infrastructure, insufficient real-world data, misleading assumptions about ease of implementation, and the need for parallel investment in system capacity. Applied to antimicrobial materials, this highlights that while biopolymer–nanoparticle composites show strong in vitro efficacy, their practical application and clinical validation remain limited, emphasizing the need for translational studies and implementation-focused research.

## 3. Multicomponent Antimicrobial Systems Based on Biopolymers and Nanomaterials

A multicomponent system is a structure in which the inclusion of two or more components achieves enhanced and/or prolonged functional effects. In such systems, biopolymer matrices—such as chitosan, alginate, carboxymethylcellulose, or gelatin are commonly employed as carriers [78,79]. These biopolymers serve not only as structural support but may also facilitate the encapsulation and controlled release of active compounds. A widely studied approach involves the incorporation of metallic nanoparticles (e.g., AgNPs, CuNPs, ZnNPs) and plant-derived bioactives (e.g., essential oils, polyphenols) into such polymeric matrices.

Multicomponent systems can be implemented through the encapsulation of active substances in nanostructures, such as nanoemulsions of essential oils with antibacterial nanoparticles, liposomes containing flavonoids and silver ions, or chitosan-based micelles and nanocapsules with the addition of peptides and phenolic compounds. These nanosystems contribute to improving the bioavailability of active substances, increasing the selectivity of their action, the possibility of targeted release (for example, depending on pH changes in the infection zone), reducing cytotoxicity and increasing storage stability [80,81,82,83].

The relevance of these multifunctional systems lies in their sustainable and efficient green synthesis, which uses plant extracts as a bio-based alternative to toxic chemicals for creating nanocomposites. Plant biomolecules like polyphenols, flavonoids, and alkaloids act as natural reducing and stabilizing agents to form uniform nanoparticles under mild conditions. When these nanoparticles are combined with biopolymer matrices like chitosan, the resulting nanocomposites exhibit enhanced stability, controlled particle size, and tunable properties suitable for applications such as catalysis, drug delivery, and antimicrobial agents [84,85].

Various types of green nanomaterials, including biogenic nanoparticles, nanocellulose, and green quantum dots, have been developed to address environmental challenges. Biogenic nanomaterials are produced using biological sources such as plants, bacteria, or fungi, offering low-cost and eco-friendly synthesis [86]. Nanocellulose exhibits excellent biodegradability and mechanical strength, while green quantum dots, made from non-toxic materials, show promising applications in bioimaging, energy, and sensing technologies [87].

Moreover, recent works have demonstrated the potential of plant-mediated synthesis—for example, the use of lemon leaf extracts to prepare g-C_3_N_4_–Cu_2_O nanocomposites with improved photocatalytic and antibacterial activities [88], or *P. granatum extract* for the green fabrication of copper nanoparticles with high electrical conductivity [89]. These approaches underline the possibility of combining renewable biological sources with nanotechnology to achieve highly functional antimicrobial properties [90].

A recent study demonstrated a green and cost-effective approach for the synthesis of gold nanoparticles (AuNPs) supported on cross-linked chitosan beads, using Solenostemma argel leaf extract as a phyto-reducing agent [91]. The chitosan matrix was cross-linked with glutaraldehyde to coordinate and stabilize Au(III) ions. Physicochemical analyses confirmed the successful incorporation of uniform spherical nanoparticles (<10 nm) into the polymer network. The resulting nanocomposite exhibited high catalytic activity, achieving 97% conversion and 99% selectivity in the oxidation of benzyl alcohol to benzaldehyde. This work highlights the potential of biopolymer-based nanocomposites synthesized with plant extracts as multifunctional and eco-friendly systems, while SEM and TEM images provide valuable insights into the uniform distribution and morphology of nanoparticles within the polymer matrix.

**Figure 4:**
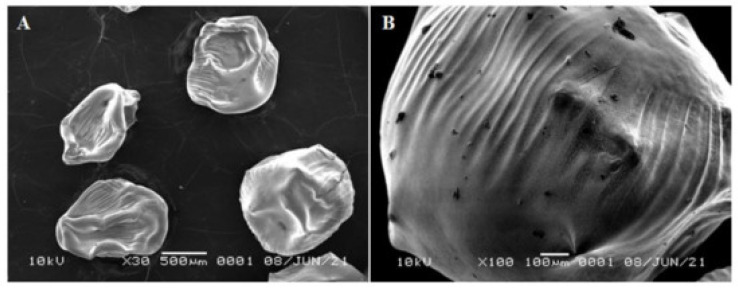
SEM spectrum of AgNP-chi-spheres using 20% NaOH, (**A**) 30×; (**B**) 100× [92]. Adapted from Mirda, E. et al. Synthesis of Chitosan-Silver Nanoparticle Composite Spheres and Their Antimicrobial Activities. *Polymers* 2021, 13, 3990. © 2021, licensed under CC BY 4.0 (https://creativecommons.org/licenses/by/4.0/ accessed on 11 November 2025).

In [92], the synthesis of silver nanoparticles–chitosan composite spheres (AgNPs-chi-spheres) was characterized using UV-Vis, FT-IR, XRD, SEM, and zetasizer nano. UV-Vis analysis showed optimal absorption at 410 nm, and XRD confirmed that the particles were crystalline and spherical. SEM analysis revealed the smallest particle size of 46.91 nm when 20% NaOH was added (Figure 4). EDX analysis showed that these particles had a smooth, non-porous structure. Zetasizer measurements indicated that the zeta potential and polydispersity index increased with higher NaOH concentrations. Microbial screening showed that AgNPs-chi-spheres with the highest NaOH concentration exhibited the largest inhibition zones against *S. aureus*, *E. coli*, and *C. albicans*, with inhibition zone diameters of 19.5, 18.56, and 12.25 mm, respectively.

Thus, multifunctional, and smart biopolymer-based systems are increasingly recognized as the next generation of antimicrobial materials, acting at the interface of biotechnology and materials science. These systems integrate bioresponsiveness, self-regulation, and targeted delivery capabilities, offering advanced control over antimicrobial performance and cytocompatibility.

## 4. Encapsulated Systems and Nanocarriers

Biologically active compounds such as metal nanoparticles (MNps) and bioactive substances of plant origin (essential oils, polyphenols) have potentially significant health benefits; however, these substances are susceptible to oxidative decomposition, which can lead to rapid release, low solubility, and poor bioavailability. The encapsulation of bioactive substances (e.g., in nanoemulsions) can protect them from the negative effects of the external environment (e.g., oxidative decomposition) and improve their physio-chemical properties, enhancing their therapeutic potential. Consequently, microencapsulation and nanoencapsulation technologies are used in the cosmetics, food, and pharmaceuticals industries, where they play a crucial role in ensuring the stability and efficacy of active ingredients [93].

Encapsulation of bioactive compounds within a matrix or protective “wall” material with the formation of microscale or nanoscale structures is widely used in the food, agricultural, pharmaceutical and cosmetic industries due to its ability to effectively protect unstable substances from the effects of adverse factors such as high temperature and oxygen, as well as to prevent the loss of volatile components, such as essential oils. Encapsulation makes it possible to create delivery systems with controlled release of the active substance, potentially improving the physio-chemical properties of products (for example, by converting liquid substances into a solid form), helps mask unpleasant tastes and odors, and increases the water solubility of difficult-to-dissolve compounds, thereby significantly expanding the possibilities of their use [94].

**Figure 5:**
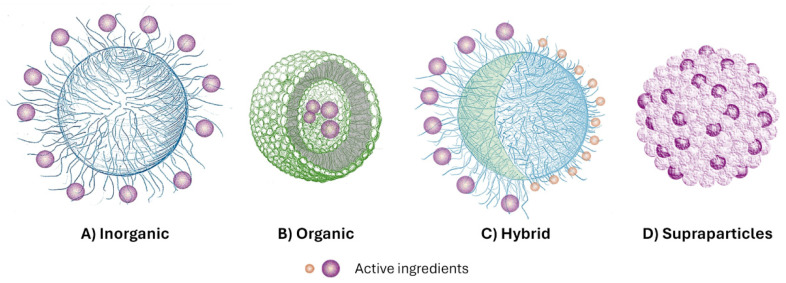
Schematic illustration of examples of: (**A**) inorganic; (**B**) organic; (**C**) hybrid nanocarriers; (**D**) supraparticles. Active ingredients (purple dots) can be covalently bound or electrostatically attached to the particle surface ((**A**) or (**C**)), encapsulated in vesicles (**B**) or trapped in the pores of nanostructured materials (**D**) [96]. Adapted from Gressler et al., *Journal of Nanobiotechnology* 2025, 23:45. © 2025, licensed under CC BY 4.0 (https://creativecommons.org/licenses/by/4.0/ accessed on 11 November 2025).

To date, research in the field of hydrophobic bioactive substance delivery highlights the key challenges associated with the development of colloidal systems that can be used in pharmaceutical preparations as carriers. Special attention is paid to the basic physio-chemical processes related to the encapsulation, stabilization, and release of bioactive components, such as solubility, phase separation, barrier properties, and mass transfer processes. Various delivery systems suitable for encapsulating hydrophilic bioactive substances are also being considered, including liposomes, multiple emulsions, fat solids, biopolymer particles, cubosomes, and biological materials-based systems. The advantages and limitations of each of these delivery systems have been reviewed [95]; likewise, nanocarriers based on the origin and chemical composition of their materials (Figure 5), as well as their size (1–1000 nm) and functional purpose, which facilitates an accurate assessment of the risks associated with the use of these systems [96].

Polymer-based nanocarriers exist in a variety of forms including micelles, vesicles, nano emulsions, and nanohydrogels, each representing potentially effective platforms for drug delivery to specific locations (e.g., cancer tumors via the enhanced permeability and retention (EPR) mechanism). These systems potentially allow both hydrophobic and hydrophilic substances to be encapsulated (ensuring their stability, protection from premature destruction), and release in the desired area.

**Figure 6:**
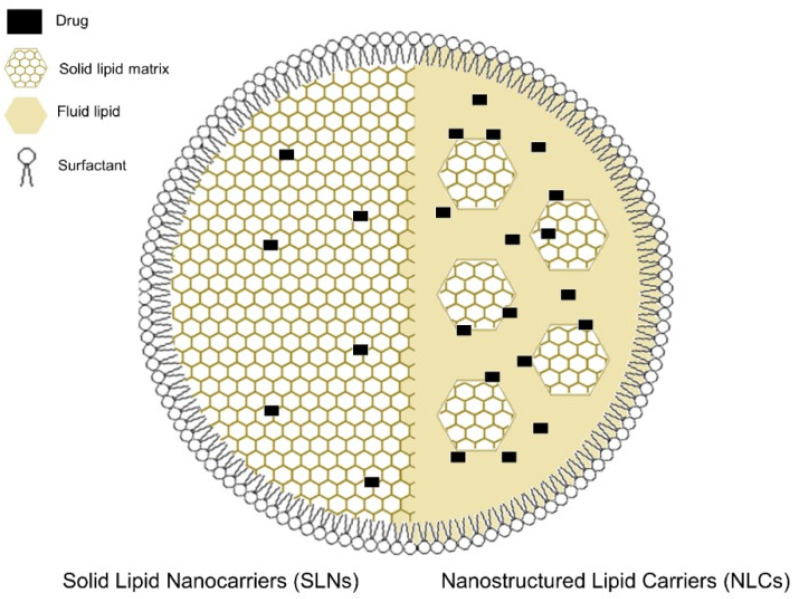
Schematic representation of the structure of a solid lipid nanoparticle (SLNs; **left half**) and a nanostructured lipid carrier (NLCs; **right half**). In SLNs, the localization of the loaded drugs is much more restricted, due to the solid lipid matrix that makes up its core, which usually translates into lower encapsulation efficiencies. The inclusion of a fluid lipid besides the solid lipid matrix in NLCs usually results in an increased drug load capacity [97]. Adapted from Makowski et al., *Pharmaceutics* 2019, 11(11), 588. © 2019, licensed under CC BY 4.0 (https://creativecommons.org/licenses/by/4.0/ accessed on 11 November 2025).

Liposomal nanocarriers, including solid lipid nanoparticles (SLN) and nanostructured lipid carriers (NLC), are actively used to transport peptides, metallic nanoparticles, and extracts, improving their stability and bioavailability [97]. SLNs consist of a solid lipid matrix stabilized by surfactants, whereas NLCs contain solid lipids combined with oil droplets and are also stabilized by surfactants. Compared to SLNs, NLCs offer higher drug loading capacity and more uniform drug release. Both systems are suitable for various routes of administration, especially for dermal applications (Figure 6).

**Figure 7:**
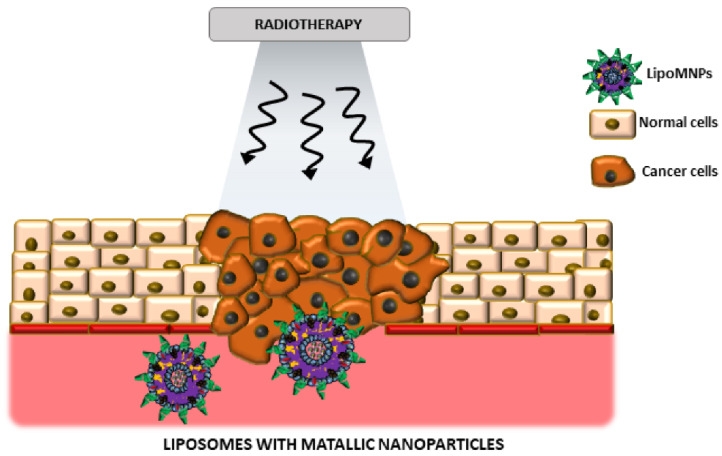
The schematic representation of LipoMNPs (Liposomes with metallic nanoparticles) usage as radiosensitizers in radiotherapy. Arrows are symbols of photons in ionizing radiation during radiotherapy [99]. Adapted from Musielak et al., *Int. J. Mol. Sci.* 2021, 22, 6229. © 2021, licensed under CC BY 4.0 (https://creativecommons.org/licenses/by/4.0/ accessed on 11 November 2025).

Liposomal and lipid nanocarriers effectively encapsulate metal nanoparticles (such as Ag, Au, or Cu), protecting them from aggregation and premature destruction [97,98]. Metal nanoparticles are actively used as agents to increase the sensitivity of cancer cells to ionizing radiation during radiation therapy. For example, gold nanorods are specifically highlighted for their unique properties that enhance radio sensitization by increasing reactive oxygen species (ROS) generation and improving treatment selectivity [98]. For interested readers we recommend an informative review of liposomal carriers, including their use in treatment and medical imaging [99]. The review emphasizes the importance of further studying the biological interactions between nanostructures and cells, which is the key to overcoming existing limitations in the clinical application of these technologies (e.g., limited understanding of biodistribution, low targeting specificity, potential long-term toxicity, and immune responses). The review also highlights the role of hybrid nanostructures, such as liposomes and metal nanoparticles, in the treatment of cancer and magnetic resonance imaging, with the possibility of using theranostic approaches. It is important that the introduction of radiosensitizers does not affect the physio-chemical properties of liposomes, and stimulating factors such as temperature, pH or light contribute to their decomposition without destroying healthy tissues (Figure 7).

Liposomes and lipid nanoparticles also serve as excellent carriers for plant extracts (for example, extracts of turmeric, tea or other natural antioxidants), improving their solubility and bioavailability. Extracts, which are often poorly soluble in water, can be encapsulated in hydrophobic regions of liposomes or SLN, which contributes to their stability and protection from oxidation. This allows the extracts to be used for various therapeutic purposes, such as antioxidant, anti-inflammatory, or antimicrobial therapy, with improved efficacy and controlled release of active substances. Thus, liposomal and lipid nanocarriers provide efficient transport of metal nanoparticles and extracts, improving their therapeutic properties, stability, and targeted delivery to the desired areas of the body.

Thus, liposomal and lipid nanocarriers provide efficient transport of metal nanoparticles and extracts, improving their therapeutic properties, stability, and targeted delivery to the desired areas of the body.

**Figure 8:**
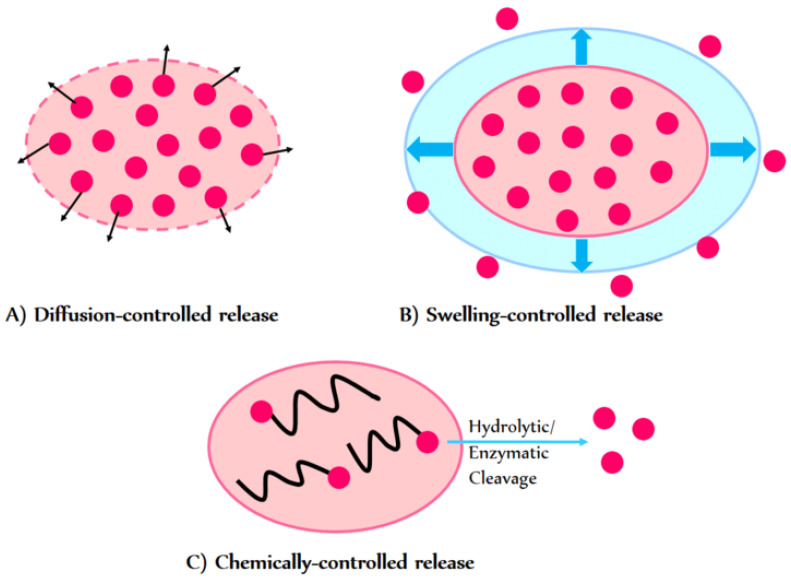
Schematic view of drug release mechanisms from hydrogels. (**A**) Diffusion-controlled release. (**B**) Swelling-controlled release. (**C**) Chemically controlled release [100]. Adapted from Ghasemiyeh, P. et al. Hydrogels as Drug Delivery Systems; Pros and Cons. Trends *Pharm. Sci. Technol*. 2019, 5, 7–24. © 2019, licensed under CC BY 4.0 (https://creativecommons.org/licenses/by/4.0/ accessed on 11 November 2025).

Alongside lipid-based carriers, biologically derived materials such as polysaccharides and proteins are increasingly explored as encapsulation matrices due to their degradability and tunable properties. In such systems, understanding the release kinetics of bioactive compounds is crucial for achieving controlled and sustained delivery. The release process is mainly governed by diffusion, polymer swelling, and degradation, often following Fickian or anomalous transport behavior. These mechanisms are commonly analyzed using models such as the Higuchi and Korsmeyer–Peppas equations, which help identify the dominant release pathways and optimize carrier performance (Figure 8) [100].

Research into the use of micelles and nanocapsules based on biopolymers for the encapsulation of active substances is also a significant area of scientific development. These approaches make it possible to effectively protect and deliver biologically active components, which is important for various fields, including the food and pharmaceutical industries. An investigation of the formation of complex coacervates formed by carboxymethylcellulose (CMC) and lactoferrin (Lf) for the encapsulation of β-carotene from Sacha inchi oil showed that the optimal conditions for the formation of coacervates are pH 5.0 and a component ratio of 1:14. Capsules obtained from these coacervates showed high encapsulation (97% of beta-carotene) and good bioavailability (67%) after digestion in an in vitro gastrointestinal model with Fickian release kinetics, highlighting the potential of using CMC complex coacervates/Lf for the protection and delivery of beta-carotene in food products [101].

Studies have also shown that cabreuva essential oil, obtained from the wood of *Myrocarpus fastigiatus*, has the potential for use in pharmaceuticals and food packaging [102]. To improve the solubility and protection of the oil, a two-stage process of forming emulsions with chitosan, SDS and PVA was proposed, followed by ion crosslinking with sodium citrate, in which the system with 0.75% chitosan and 1% SDS proved to be the most stable. As an alternative to essential oil encapsulation, the electrospinning method was used, which made it possible to create nanofibers that effectively control the release of oil with demonstrable activity against a variety of microorganisms (*Candida albicans*, *Escherichia coli*, *Staphylococcus aureus* and *Staphylococcus epidermidis*).

Thus, the encapsulation of active compounds using biopolymers, nanoemulsions, liposomes, micelles, and other nanostructured carriers have significant potential for applications in pharmaceuticals, the food industry, and other fields. However, for the effective and safe use of these delivery systems, additional research is needed to improve their properties and optimize them.

## 5. Biotechnological Approaches: Microorganisms for the Creation of Antimicrobial Biopolymers

In recent decades, biotechnologies have made significant progress in the development of innovative methods for the production of antimicrobial materials, including biopolymers. One of the most promising approaches is the use of microorganisms for the synthesis of biopolymers with antimicrobial activity [103,104,105,106]. Microorganisms such as bacteria, fungi, and algae are natural sources for the synthesis of bioactive compounds. These organisms can produce biopolymers that have antimicrobial properties, contributing to the creation of new materials for medical, pharmaceutical, and environmental applications [107].

Modern research is aimed at identifying and optimizing microorganisms capable of producing such biopolymers, as well as understanding the mechanisms of their antimicrobial activity. As a result, new ways of using antimicrobial biopolymers are being developed in the fields of medicine, packaging, pollution protection and other fields [108,109]. It is possible to purify bacterial cellulose (BC) produced by *Komagataeibacter xylinus* and generate BC-based biomaterials (e.g., films) and improve the mechanical properties of these films by immersion in solutions of polyvinyl alcohol and chitosan [110]. Parameters such as moisture content, mechanical properties, vapor permeability, as well as morphology and structural characteristics were evaluated using various analytical methods. The results showed that the addition of chitosan significantly increases the strength of the films, reaching values similar to synthetic polymer films. Also, the addition of polyvinyl alcohol reduced the permeability to water vapor. The developed films have protective properties against ultraviolet radiation and an optimal appearance, which opens up prospects for their application in various fields, including medical and packaging technologies.

Biomaterials in which biopolymers are integrated with metal nanoparticles (for example, Ag, Cu or Zn) and plant extracts. Metal nanoparticles, due to their powerful antimicrobial properties, provide active protection against pathogens, while plant extracts containing phytochemical compounds can enhance the antimicrobial effect and contribute to additional biological activity. This combination of natural and synthetic components has prospects for the development of sustainable and environmentally friendly materials with a wide range of applications in medicine, pharmaceuticals, and the food industry [111,112]. The antibacterial and antioxidant properties of chitosan (CS) nanoparticles with the addition of copper oxide (CuONPs) and grape seed extract (GSE) were studied [113]. The nanocomposites effectively absorb the DPPH radical (74.2% at 100 µg/mL), which is comparable to ascorbic acid, and have pronounced antibacterial activity. The CS/CuO/GSE composites demonstrated inhibitory activity against *E.* (9 mm inhibition zone) and *Streptococcus mutans* (14 mm inhibition zone) at a concentration of 100 µg/mL. The minimum inhibitory concentrations were 312 µg/mL for *S. mutans* and 5 mg/mL (5000 µg/mL) for *E. coli*. Thus, the developed nanocomposite has a high potential for use in the fight against resistant bacteria, as well as an antioxidant.

Modern methods for modifying biodegradable polymers using chemical elements, microorganisms, and enzymes to enhance their antibacterial activity were reviewed [114], covering the processing of biopolymers, the creation of composites and the modeling of their properties, which contributes to improving efficiency in the fight against microbial pollutants. The issues of environmental safety of these materials and their disposal without harm to the environment were also considered.

## 6. Current Issues and Challenges

Despite the progress in the modification of biodegradable polymers, modern methods of chemical modification, as well as the use of microorganisms and enzymes, require further improvement [115]. Under real-world operating conditions, it is often not possible to achieve the necessary stability and durability of antibacterial properties [116], which underscores the need to develop more effective and versatile methods.

From the point of view of environmental and economic aspects regarding polymer production/disposal there are challenges [117], likewise with their current costs [118].

**Figure 9:**
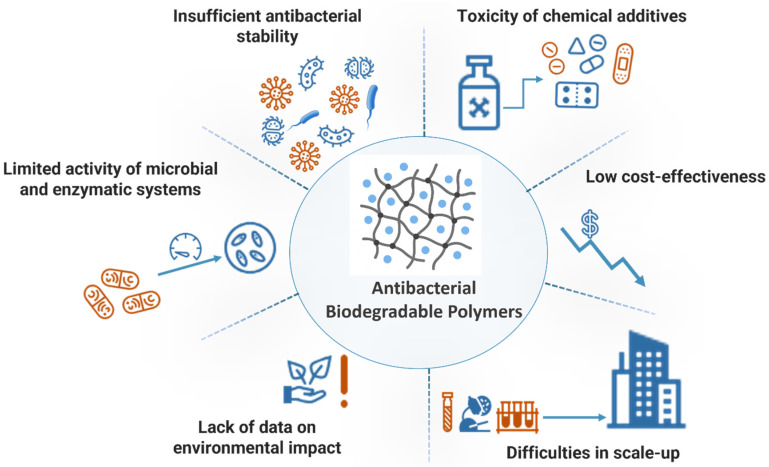
Current problems and challenges related to the development and application of antibacterial biodegradable polymers.

The interaction of new polymers with the environment (particularly understanding of the long-term effects of application of such polymers on the ecosystem and the biosphere) is an area of ongoing research [119]. Thus, despite significant progress, the industry is facing several challenges (Figure 9), the solution of which will require interdisciplinary research approaches.

## 7. Prospects for the Development of Antimicrobial Biopolymer Matrices

The problem of antimicrobial resistance and the need to develop safe, environmentally sustainable and highly effective materials stimulates research into the creation of biopolymer matrices with improved antimicrobial properties. In the future, this field will be developed using new approaches aimed at creating materials that will not only effectively fight infections, but also ensure environmental safety [120].

The introduction of metal nanoparticles will open new opportunities for the creation of medical and pharmaceutical products such as antiseptic coatings, implants and bandages for wound healing, minimizing the risk of infection and accelerating regeneration processes. Several clinical products incorporating metal nanoparticles are currently available [121,122,123]. For example, Atrauman Ag is a silver-impregnated tulle dressing, providing antibacterial activity and widely used for the management of acute and chronic wounds. A summary of commercially available hydrogels and dressings containing metal nanoparticles is presented in Table 1.

Several novel products are either currently being evaluated or have already been assessed in clinical studies. Hydrogels containing metal nanoparticles have exhibited promising antimicrobial activity and favorable wound healing performance outcomes [124,125,126,127,128]. The use of plant extracts for the synthesis of metal nanoparticles and their integration into biopolymer matrices will not only improve antimicrobial properties, but also increase environmental safety, making materials more natural and safer for use in cosmetology and pharmaceuticals. Plant extracts with activity can become part of complex formulas, opening up new perspectives in the development of natural therapeutic and preventive agents [129,130]. Innovative technologies such as encapsulation and the use of nanocarriers will play a key role in the fight against microbial infections. These technologies will make it possible to create devices with a long service life, effectively controlling the release of antimicrobial agents, which will significantly increase their effectiveness, especially in medical devices and patches [131,132,133]. The transition to biodegradable biopolymers and the use of environmentally friendly components will help protect the environment, reducing the negative impact on nature and minimizing pollution. Modified biopolymers with improved antimicrobial properties will be widely used in the medical and pharmaceutical industries, as well as in areas such as the food industry, agriculture and environmental protection. Their use is especially relevant for the protection of medical devices, the delivery of medicines and the removal of microbial contamination [134]. Future advances in antimicrobial biopolymers will be closely linked to the development of nanotechnology, biotechnology, materials science, and environmental sciences. An interdisciplinary approach will form the basis for creating innovative solutions combining microbiological safety, environmental friendliness and high efficiency [135].

Cochrane reviews are evidence-based healthcare reviews, which are reliable and based on independent evidence to inform healthcare decisions for professionals, patients, and policymakers worldwide. There are currently 134 Cochrane reviews featuring the keyword antimicrobial for the treatment of a variety of conditions (associated with infections, devices, e.g., catheters, surgeries, etc.), and sales practices to address issues like antimicrobial resistance [136]. The conclusion of the Cochrane reviews was that there is currently insufficient evidence from randomized control trials to suggest that hydrogel dressings are effective in treating foot/leg ulcers or indeed as skin grafts [137], likewise there is insufficient evidence to recommend the use of silver-containing dressings or topical agents for treatment of infected or contaminated chronic wounds or diabetic foot ulcers [138]. Clearly, if such biomaterials are to find real world applications, there is a need for further well-designed systematic studies in the future.

## 8. Conclusions

The creation of biopolymer matrices with improved antimicrobial properties is a promising direction in the fight against infections and microbial contamination. The development of these technologies, supported by innovative methods and interdisciplinary research, will open up wide opportunities for the use of such materials in medical, pharmaceutical, and environmental practice, providing not only effective treatment, but also support for sustainable development in various industries.

Recent advances demonstrate that the incorporation of natural antimicrobial agents, such as metal nanoparticles and plant-derived extracts, can significantly enhance the functional performance of biopolymers. These hybrid systems combine the advantages of biodegradability, safety, and environmental friendliness with strong antimicrobial efficacy, making them attractive alternatives to conventional synthetic materials. However, challenges remain in achieving controlled release, maintaining stability of active components, and ensuring the reproducibility of antimicrobial activity across different systems. Recent systematic reviews indicate that antimicrobial efficacy observed in controlled laboratory studies frequently does not translate reliably to real-world applications, largely due to experimental variability and the absence of standardized assessment protocols, underscoring the necessity for rigorous in vivo validation and translational studies.

Future research should therefore focus on optimizing composition–structure–function relationships to balance antimicrobial efficiency, mechanical performance, and biocompatibility. Moreover, attention should be given to regulatory compliance, for example, comprehensive biocompatibility testing, via the implementation of standardized antimicrobial assays (e.g., ISO or ASTM protocols). Addressing these aspects will facilitate the safe translation of laboratory results into real-world applications and accelerate the development of next-generation antimicrobial biopolymer materials.

## Figures and Tables

**Table 1:** Commercially available metal nanoparticle-containing hydrogels and dressings.

Product Name	Metal	Characteristics	Source
Atrauman Ag Antibacterial Silver Dressing	Ag	Silver-impregnated tulle dressing; antibacterial on contact, prevents maceration, conforms to wound; infected or colonized wounds, prophylaxis	https://medicaldressings.co.uk/atrauman-ag-antibacterial-silver-dressing/ (accessed on 11 November 2025)
Acticoat Flex 3	Ag	Flexible silver nanoparticle dressing; burns, surgical wounds	https://medicaldressings.co.uk/acticoat-flex-3-silver-coated-antimicrobial-dressings/ (accessed on 11 November 2025)
Mepilex Border Ag Dressing	Ag	Silver-impregnated foam dressing; supports moist wound healing, reduces microbial load, suitable for chronic and post-operative wounds	https://medicaldressings.co.uk/mepilex-border-ag-silver-antimicrobial-foam-dressing/ (accessed on 11 November 2025)
Suprasorb^®^ A + Ag	Ag	Antimicrobial calcium-alginate dressing with silver ions; high exudate absorption; forms gel upon contact with the wound; suitable for infected and exuding wounds.	https://lohmann-rauscher.co.uk/products/woundcare/suprasorb-range/supersorb-a-ag (accessed on 11 November 2025)
MedCu^®^ Copper Oxide Dressing	Cu	Hydrocolloid with copper nanoparticles; chronic wounds, diabetic foot	https://medcu.com/medcu-2/ (accessed on 11 November 2025)
AQUACEL^®^ Ag SURGICAL cover dressing	Ag	Combination hydrocolloid/Hydrofiber^®^ dressing with ionic silver; manages serosanguinous fluid; PU film provides viral, waterproof, and bacterial barrier	https://www.convatec.com/en-gb/products/advanced-wound-care/wound-type/pc-wound-closed-surgical-solutions/aquacel-surgical-cover-dressing/ (accessed on 11 November 2025)
ACTISORB SILVER 220	Ag	Activated charcoal + silver; antimicrobial, absorbs toxins and odor; partial/full-thickness wounds, ulcers, burns, donor sites, surgical wounds	https://medicaldressings.co.uk/actisorb-silver-220-activated-charcoal-dressing/ (accessed on 11 November 2025)
SilvaSorb Silver Antimicrobial Wound Gel	Ag	Silver-releasing hydrogel; broad-spectrum antimicrobial, moist wound healing; pressure wounds, ulcers, burns, surgical wounds, donor sites	https://www.woundsource.com/product/silvasorb-gel (accessed on 11 November 2025)
